# Identifying influential nodes based on network representation learning in complex networks

**DOI:** 10.1371/journal.pone.0200091

**Published:** 2018-07-09

**Authors:** Hao Wei, Zhisong Pan, Guyu Hu, Liangliang Zhang, Haimin Yang, Xin Li, Xingyu Zhou

**Affiliations:** College of Command Information System, Army Engineering University of PLA, Nanjing, China; Universidad Rey Juan Carlos, SPAIN

## Abstract

Identifying influential nodes is an important topic in many diverse applications, such as accelerating information propagation, controlling rumors and diseases. Many methods have been put forward to identify influential nodes in complex networks, ranging from node centrality to diffusion-based processes. However, most of the previous studies do not take into account overlapping communities in networks. In this paper, we propose an effective method based on network representation learning. The method considers not only the overlapping communities in networks, but also the network structure. Experiments on real-world networks show that the proposed method outperforms many benchmark algorithms and can be used in large-scale networks.

## 1. Introduction

Identifying influential nodes in complex networks has gained great attention in the research community [1–7]. In recent years, many methods have been put forward to find influential nodes in complex networks. The knowledge of node’s spreading ability shows new insight for application such as controlling propagation of messages and rumors in social networks[8], ranking reputation of scientists[9] and finding social leaders [10],etc.

The early measure of identifying influential nodes proposed by Shimbel is Stress Centrality[11] in 1950s. He suggested that the centrality of a node should be the total number of shortest paths that go through it. Degree Centrality[12] is a direct and effective method to measure the importance of nodes, but it neglects the global structure of the network. Eigenvector Centrality[13] considers the importance of node’s neighbors. Betweenness Centrality[14] and Closeness Centrality[15] need to know all topology information of networks in advance and cannot be applied to large-scale networks. Comin et al.[16] combined degree and betweenness, but it is a time-consuming measure. Chen et al.[2] proposed a semi-local centrality measure, which is a tradeoff between the low-relevant degree centrality and other time-consuming measures. Additionally, Chen et al.[17] proposed ClusterRank, a local ranking method that considers the clustering coefficient of a node. Kitsak et al.[1] suggested that the influence of a node is mainly dependent on its position in the network and proposed K-shell to measure the importance of a node. However, K-shell considers only the links between the residual nodes, whereas the links that connect to the exhausted nodes are entirely ignored. Johanhyun Bae et al.[18] extended the K-shell and proposed C_*nc*_ and C_*nc*+_. Zeng et al.[6] proposed a mixture decomposition method called Mixed Degree Decomposition (MDD), which considers both the residual degree and the exhausted degree. FD Malliaros et al.[19] proposed K-truss decomposition and suggested that the topological properties of the nodes play a crucial role. Lü et al.[20] showed the relationship between degree, H-index and coreness by constructing an operator and proved that the convergence to coreness can be guaranteed even under an asynchronous updating process. Numerical analyses in real networks suggested that the H-index is a good tradeoff that can better quantify node influence than either degree or coreness. In the same year, Lü et al.[21] reviewed the vital nodes identification methods and experimented on real-world networks to compare the mainstream algorithms. The methods of identifying influential nodes based on random walk are mainly used in web page sorting. The typical methods are Kleinberg's HITS algorithm[22], Google's PageRank algorithm[23] and Lv's LeaderRank algorithm[8].

Most of the previous methods only consider the node's topology information. In fact, real-world networks often have a strong community structure[24]. In social as well as other types of networks, nodes often belong to multiple communities simultaneously[25]. Influential nodes always act as "bridging" between the communities and exist in community overlaps. In this paper, we propose a new local central method to identify the influential nodes. The method assumes that the more communities a node belongs to, the greater influence of the node. To identify the influential nodes, we use the network representation learning to detect overlapping communities, and then combine with the topology information of the nodes. Experiments show state of the art performance in terms of the quality of identified influential nodes.

## 2. Method

### 2.1 Network representation learning model

Network representation learning aims at learning distributed vector representation for each vertex in a network. It is also increasingly recognized as an important aspect for network analysis. Network representation learning tasks can be broadly abstracted into the following four categories: (a) node classification[26], (b) link prediction[27], (c) clustering[28], and (d) visualization[29].

**Fig 1:**
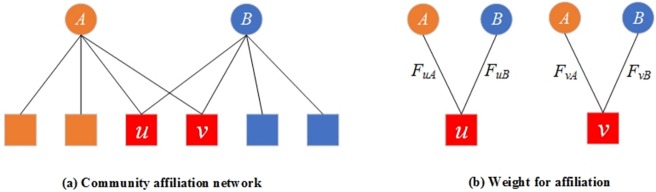
Bipartite community affiliation graph.

J. Yang et al.[25] proposed the BIGCLAM model for network representation learning, which also covers the overlapping community detection. The model assumes that the overlaps of communities tend to be more densely connected than the non-overlapping parts. We briefly introduce this model with a bipartite graph in Fig 1. In Fig 1, the circles on the top represent communities, the squares at the bottom represent the nodes of the graph, and the edges indicate node community affiliations. Each affiliation edge in the bipartite affiliation network has a nonnegative weight. The higher the node’s weight to the community the more likely is the node to be connected to other members in the same community. Each community *c* creates an edge between nodes *u* and node *v* with probability 1 − *exp*(−*F*_*uc*_ ∙ *F*_*vc*_). Where *F*_*uc*_ is the nonnegative weight of node *u* to community *c*. The higher the value of *F*_*uc*_, the more likely is the node *u* has an edge with the nodes in community *c*. Furthermore, the model assumes that each community creates edges independently. For example, in Fig 1(A), node *u* and node *v* belong to community *A* and community *B* simultaneously. In Fig 1(B), *F*_*uA*_ and *F*_*uB*_ indicate the node *u*’s weight of the affiliation to the community *A* and community *B* respectively. In community *A*, the probability of existing an edge between node *u* and node *v* is 1 − *exp*(−*F*_*uA*_ ∙ *F*_*vA*_). Similarly, the probability that there is an edge between node *u* and node *v* is 1 − *exp*(−*F*_*uB*_ ∙ *F*_*vB*_). Note that since node *u* and node *v* belong to community *A* and community *B* simultaneously, node *u* and *v* receive two chances to create a link. As each community creates edges independently, the probability of an edge existing between node *u* and node *v* is 1 − *exp*(−∑_*c*∈*A*,*B*_*F*_*uc*_ ∙ *F*_*vc*_).

Given a network *G*(*V*,*E*), where *V* is the node set and *E* is the edge set. Let $F\in R^{N\times K}$ be a nonnegative matrix, where *N* is the number of nodes and *K* is the number of communities. *F*_*uc*_ is the weight between node *u* ∈ *V* and community *c* ∈ *K*. Given *F*, BIGCLAM generates *G*(*V*,*E*) by creating edge (*u*,*v*) ∈ *E* between a pair of nodes *u*,*v* ∈ *V* with probability *p*(*u*,*v*):

$$p(u,v)=1-exp(-F_{u}∙{F_{v}}^{T}),$$ (1)

where *F*_*u*_ is a weight vector for node *u*. Each element in *F*_*u*_ is the weight of node *u* to the corresponding community. The model aims to finding the most likely affiliation factor matrix $\hat{F}\in R^{N\times K}$ of the underlying network *G* by maximizing the likelihood:

$$\hat{F}=argmax\;P(G|F),$$ (2)

where

$$P(G|F)=\prod_{(u,v)\in E}p(u,v)\prod_{(u,v)\notin E}\left(1-p(u,v)\right).$$ (3)

Many times, we take the logarithm of the likelihood and call it log- likelihood:

$$\hat{F}=argmax\log P(G|F),$$ (4)

where

$$logP(G|F)=\sum_{(u,v)\in E}log(1-exp(-F_{u}F_{v}^{T}))-\sum_{(u,v)\notin E}F_{u}F_{v}^{T}.$$ (5)

BIGCLAM learns a *K*-dimensional non-negative vector for each node in the network by optimizing the problem of Eq 4. Each dimension in the vector represents the probability that the node belongs to the corresponding community. After learning $\hat{F}$, the model need to determine whether node *u* belongs to community *c* or not from the value of *F*_*uc*_. It ignores the membership of node *u* to community *c* if *F*_*uc*_ is below some threshold *δ*. Otherwise (*F*_*uc*_ > *δ*), it regards *u* as belonging to *c*. One node can belong to more than one community simultaneously. Based on the BIGCLAM model, we assume that the nodes in the community overlaps play the ‘bridging’ role between the communities. As these nodes belong to multiple communities, information through these nodes can be easily spread to other communities. It is reasonable to assume that nodes in community overlaps have greater influence.

### 2.2 Network constraint coefficient

**Fig 2:**
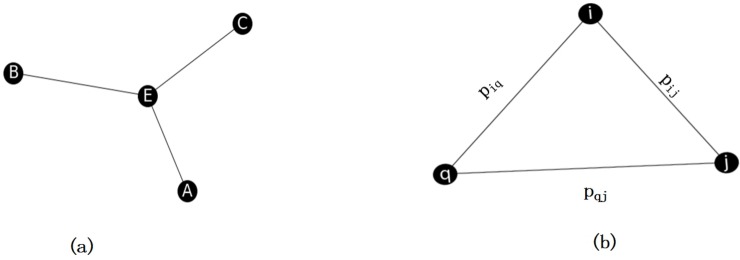
The concept of structural holes.

Structural holes is a concept from social network research, which is originally developed by Burt[30][31]. A structural hole is understood as a gap between two individuals who have complementary sources to information. Fig 2(A) is a structural hole of node E. The position of node E makes it serve as a bridge or a ‘broker’ between three different nodes. Thus, node E is likely to receive some non-redundant information from its contacts. The term ‘structural holes’ is used for the separation between non-redundant contacts. Because of the hole between two contacts, they provide network benefits to the third party (to node E).

Burt used the network constraint coefficient *C* to measure the constraints imposed by forming a structural hole:

$$C_{i}=\sum_{j\in \Gamma (i)}(p_{ij}+\sum_{q\epsilon (\Gamma (j)\cap \Gamma (i))}(p_{iq}p_{qj}{))}^{2},$$ (6)

where *Γ*(*i*) is the neighbor set of node *i*. As shown in Fig 2(B), *p*_*ij*_ is proportion of *i’*s energy invested in relationship with *j* and $p_{ij}=\frac{1}{N(i)}$, where *N*(*i*) represents the degree of node *i*. $p_{iq}=\frac{1}{N(i)}$ and $p_{qj}=\frac{1}{N(q)}$ represent node *i’*s and node *j’*s energy respectively invested in relationship with the common neighbor *q*. From Eq 6 we can see that a node with a small constraint coefficient indicates that the degree of the node is large and the connections among neighbors are sparse. Thus, the node with a small constraint coefficient would have more chances to spread the information to a large portion of the network. The smaller the constraint coefficient of a node is, the faster the node can spread information.

### 2.3 Ranking method

Nodes in community overlaps play the ‘bridging’ role between the communities. Information can be spread to multiple communities through these nodes. The number of communities a node be longs to can be regarded as its propagation capacity. The more communities a node belongs to, the more communities the node can influence. Network constraint coefficient of a node can be regarded as the propagation speed in community. The smaller the constraint coefficient of a node is, the faster the node can spread information. We consider both propagation capacity and propagation speed of a node to evaluate its influence, which denoted by OC. The OC of node *i* is defined as follows:

$${OC}_{i}=\frac{\sum_{j\in \Gamma (i)}\sum_{k\epsilon \Gamma (j)}10^{-C_{k}}*Nb(k)}{\max OC},\;i=1,2,3\ldots N,$$ (7)

where *Γ*(*i*) is the neighbor set of node *i*, *C*_*k*_ is the network constraint coefficient of node *k*, *maxOC* is the normalization factor, *Nb*(*k*) represents the total number of communities that node *k*'s neighbors belong to. For example, assuming that node *i* has 3 neighbors *a*, *b*, *c*, node *a* has 2 communities named 1, 4, node *b* has 3 communities named 1, 2, 3, and node *c* has 4 communities named 2, 4, 5, 6, then the communities that the neighbors of node *i* have are 1, 2, 3, 4, 5, 6. Thus, *Nb*(*i*) = 6.

To identify influential nodes in the network, we need to know the total number of communities that each node belongs to and their constraint coefficients. First, we use the BIGCLAM model to detect overlapping communities, and calculate the total number of communities that each node belongs to. Second, we calculate the network constraint coefficient for each node based on Eq 6. Third, according to Eq 7, we calculate the OC value of each node. Note that if there are no overlapping communities in the network, OC degrades to the network constraint coefficient.

## 3. Experiments

### 3.1 Evaluation method

To evaluate the performance of the proposed method, we use the SIR model[32] to examine the influence of nodes. The model is used to simulate the spread of the virus or information process. The SIR model divides the network nodes into three types: (1) Susceptible nodes, healthy but not immune and can be infected; (2) Infected nodes, already infected and can infect susceptible nodes; (3) Recovered nodes, which have been cured and cannot be infected again. In the beginning, the node to be tested is in the Infected state whereas the rest of the nodes of the network are in the Susceptible state. This node triggers a spreading process where every infected node can infect its neighbors at each timestep *t* with probability *β*. Each infected node is cured with probability *γ*. In this paper, we set *γ* = 1, which means that each node has only one chance to infect its neighbors in every round. The sum (*F*(*t*)) of recovered nodes at time *t* when there is no infected node exiting in the network are defined as the influence of the node. In order to ensure the spreading process, we set *β* to be slightly larger than the epidemic threshold ($\beta_{th}=\frac{\langle k\rangle }{\left[\langle k^{2}\rangle -\langle k\rangle \right]}$) in the network[33], where 〈k〉 and 〈k^2^〉 denote the average degree and the second order average degree, respectively. In this paper, we set $\beta_{th}=\frac{\langle k\rangle }{\langle k^{2}\rangle }$.

To quantify the correctness of the ranking methods, we adopt Kendall’s tau[34] as a rank correlation coefficient, which is defined as follows:

$$\tau (R_{1},R_{2})=\frac{n_{c}-n_{d}}{\sqrt{{(n}_{t}-n_{t1})(n_{t}-n_{t2})}},$$ (8)

where *R*_1_ and *R*_2_ are two different rank lists, *n*_*t*_ = *n*(*n* − 1)/2, *n*_*t*1_ = ∑_*i*_*t*_*i*_(*t*_*i*_ − 1)/2, *n*_*t*2_ = ∑_*j*_*t*_*j*_(*t*_*j*_ − 1)/2, *t*_*i*_ and *t*_*j*_ are the number of tied values in the *i*th and jth groups of ties, respectively. *n*_*c*_ and *n*_*d*_ are the numbers of concordant and discordant pairs, respectively. For example, let *X* and *Y* be two ranking lists. (*x*_1_, *y*_1_), (*x*_2_, *y*_2_),…,(*x*_*i*_, *y*_*j*_) are a set of joint ranks from *X* and *Y*, respectively. Any pair of ranks (*x*_*i*_, *y*_*i*_) and (*x*_*j*_, *y*_*j*_) is said to be concordant if *x*_*i*_ > *x*_*j*_ and *y*_*i*_ > *y*_*j*_ or *x*_*i*_ < *x*_*j*_ and *y*_*i*_ < *y*_*j*_. If *x*_*i*_ > *x*_*j*_ and *y*_*i*_ < *y*_*j*_ or *x*_*i*_ < *x*_*j*_ and *y*_*i*_ > *y*_*j*_, the pair is said to be discordant. If *x*_*i*_ = *x*_*j*_ or *y*_*i*_ = *y*_*j*_, the pair is neither concordant nor discordant. This metric quantifies the similarity between the orderings of the measures and the real ranking.

### 3.2 Experimental data

Nine real-world networks are used to evaluate the performance of the proposed method: (1) all meeting articles that appeared in 1994-2000(**GDciting**). The data can be obtained on *“https://www.aminer.cn/citation”*; (2) US airport flights(**USAir97**). The data can be downloaded on “*http://vlado.fmf.uni-lj.si/pub/networks/data/*”; (3) collaboration network of scientists(**Netscience**)[35]; (4) communication network of Blogs(**Blogs**)[36]; (5) an e-mail communication network(**Email**)[37]; (6) C.elegans networks(**C.elegans**)[38]; (7) Lusseau’s Bottlenose Dolphins(**Dolphins**)[39]; (8) Arxiv COND-MAT collaboration network(CA-CondMat)[40]; (9) Amazon network data(**Amazon**). The data can be downloaded on *“https://snap.stanford.edu/”*. Table 1 shows the information of each network, where *n* is the number of nodes, *m* is the number of edges, *C* is the number of communities divided by the BIGCLAM model, *MLC* is the maximum number of communities owned by the node in the network.

**Table 1:** The statistical properties of the networks, where *n* is the number of nodes, *m* is the number of edges, C is the number of communities divided by BIGCLAM model, MLC is the maximum number of communities the nodes have in the network.

****Network****	*n*	*m*	*C*	*MLC*
****GDciting****	311	647	13	4
****USAir97****	332	2126	17	10
****Netscience****	1461	2742	96	5
****Blogs****	112	425	8	3
****Email****	1133	5451	39	10
****C.elegans****	248	468	13	4
****Dolphins****	62	159	7	2
****CA-CondMat****	23133	93497	100	34
****Amazon****	334863	925872	73854	139

### 3.3 Experimental results

In this section, we compare the proposed method OC with Degree Centrality(DC), Betweenness Centrality(BC), Closeness Centrality(CC), Eigenvector Centrality(EC), *C*_*nc*_, C_nc+_, Network Constraint Coefficient(NC) and K-shell(KS). In each implementation, one node is selected to be infected, and then infects its neighbors according to the SIR model. The influence of the node (*F*(*t*)) is the sum of recovered nodes when the spreading process fade out. This value represents the average over multiple executions of the model (we performed 1000 simulations for large and 100 simulations for small datasets). Without special explanation, in this paper, the value of *β* is shown in Table 2, *γ* = 1, and the threshold for the dataset is 1000 nodes.

**Table 2:** The ranking results of each network. **Here *β***_***th***_ **is the epidemic threshold for networks; *β* is the infection probability in SIR simulation; *τ*(∙) represents the Kendall correlation coefficient of corresponding methods for given *β*. “-” means the method is still no result when running time exceeds 48 hours**.

Network	*β*_*th*_	*β*	*τ*_*DC*_	*τ*_*BC*_	*τ*_*CC*_	*τ*_*NC*_	*τ*_*KS*_	$\tau_{C_{nc}}$	$\tau_{C_{nc+}}$	*τ*_*EC*_	*τ*_*OC*_
**GDciting**	0.102	0.176	0.71	0.52	0.82	0.71	0.70	0.84	0.85	0.89	**0.95**
**USAir97**	0.021	0.033	0.85	0.60	0.84	0.88	0.84	0.94	0.94	0.93	**0.96**
**Netscience**	0.115	0.264	0.63	0.20	0.81	0.75	0.57	0.72	0.73	0.88	**0.89**
**Blogs**	0.067	0.106	0.86	0.69	0.88	0.82	0.78	0.91	0.92	**0.94**	0.92
**Email**	0.05	0.079	0.74	0.61	0.77	0.75	0.75	0.80	0.81	0.84	**0.87**
**C.elegans**	0.143	0.204	0.72	0.57	0.84	0.70	0.70	0.83	0.83	0.85	**0.93**
**Dolphins**	0.147	0.231	0.72	0.52	0.70	0.76	0.56	0.79	0.80	0.75	**0.97**
**CA-CondMat**	0.045	0.054	0.32	0.25	0.35	0.32	0.12	0.48	0.26	0.64	**0.66**
**Amazon**	0.095	0.114	0.43	-	-	0.46	0.23	0.52	0.53	0.56	**0.61**

In Table 2, we compare the Kendall correlation coefficient *τ* of different ranking methods. The results in Table 2 manifest that our method outperforms the other methods in most cases.

**Fig 3:**
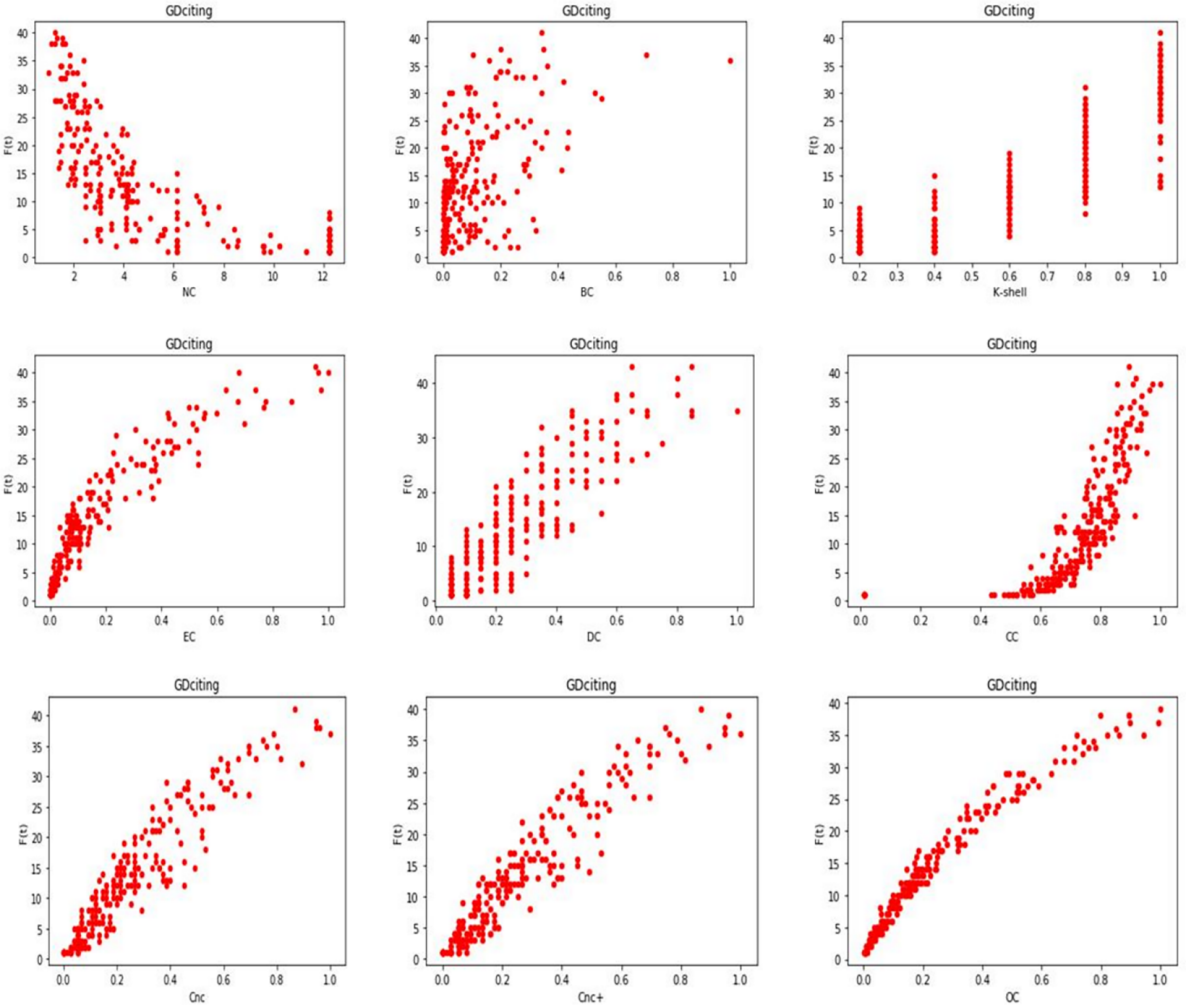
The relation between node’s influence and the ranking methods in GDciting.

**Fig 4:**
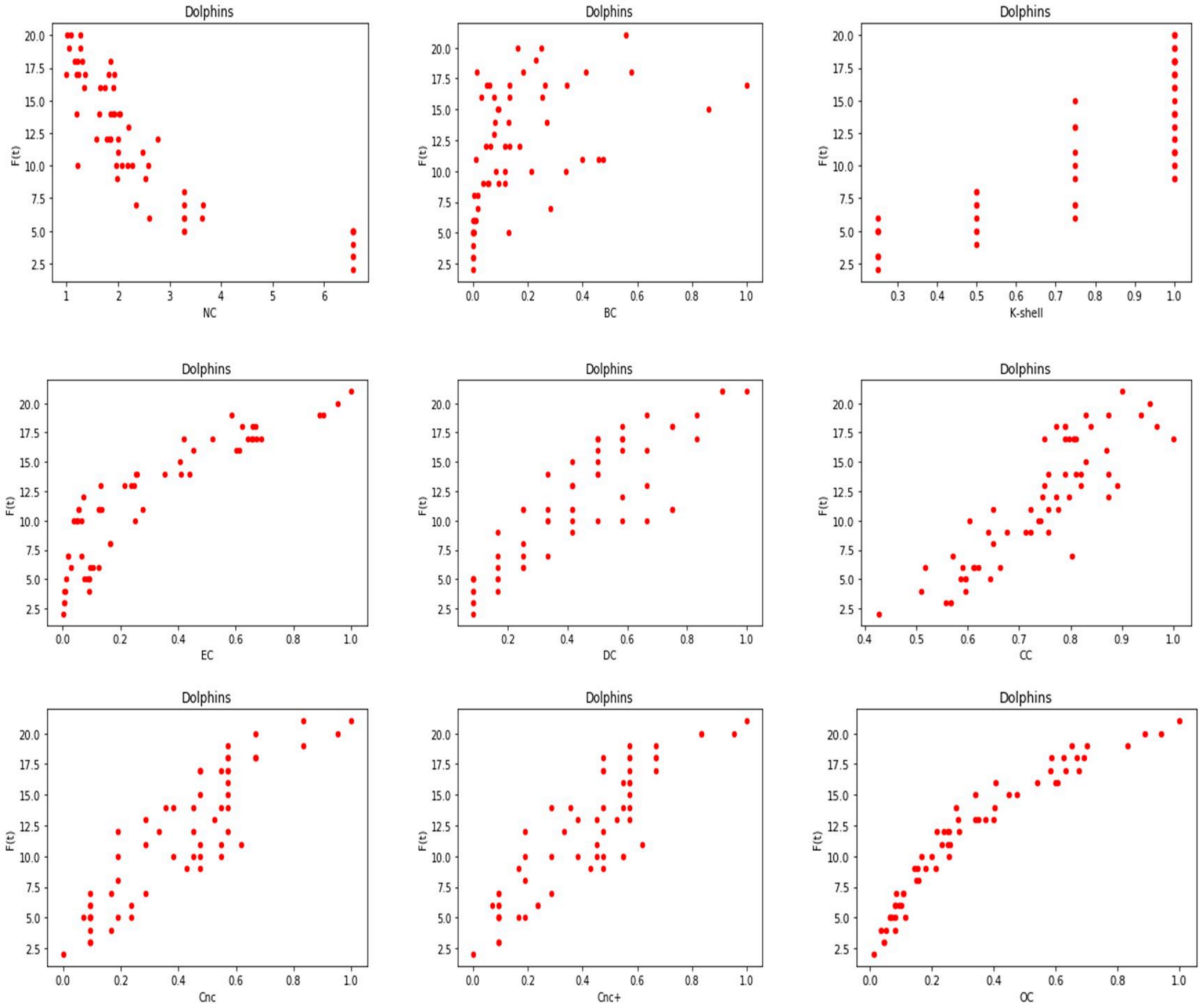
The relation between node’s influence and the ranking methods in Dolphins.

**Fig 5:**
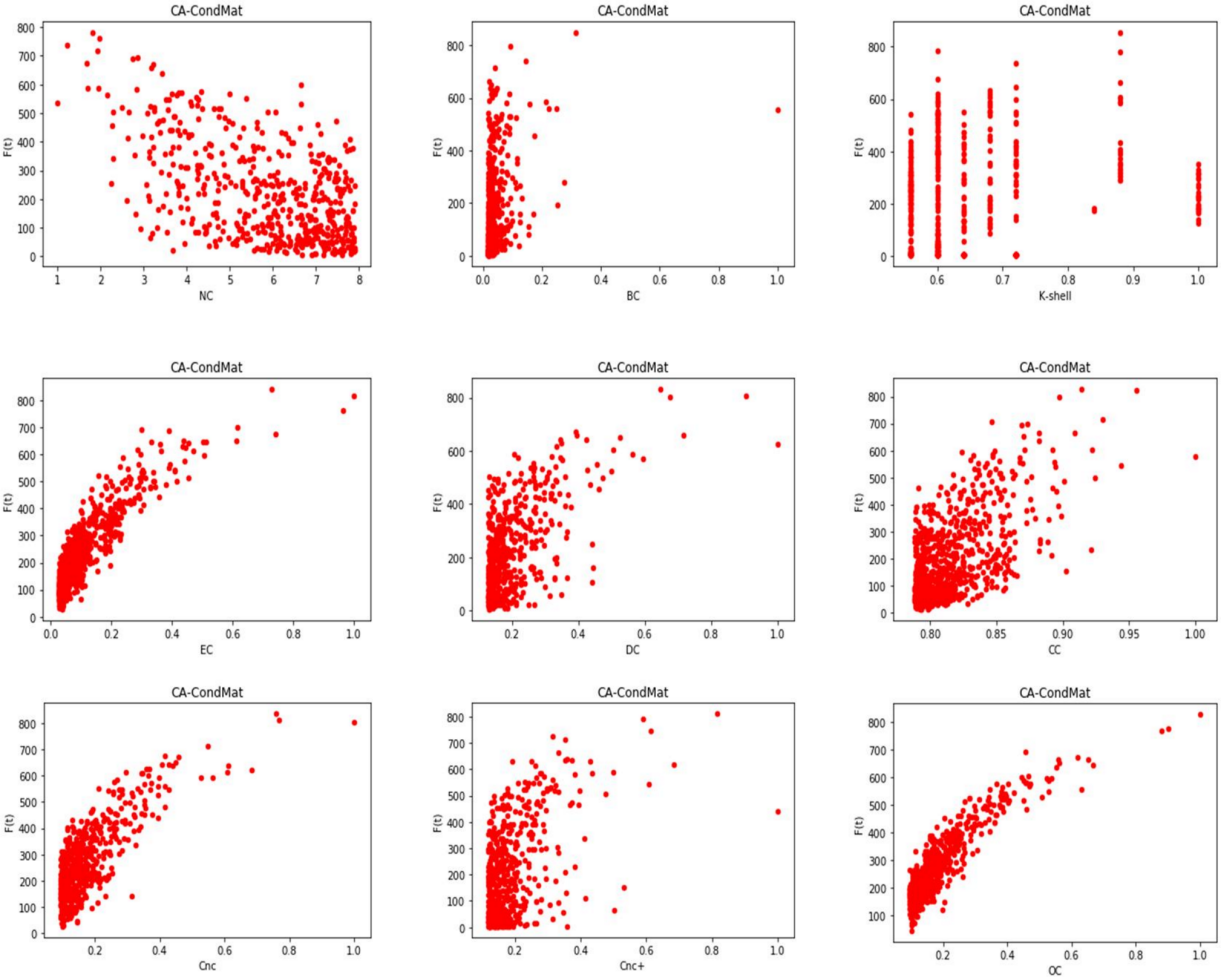
The relation between node’s influence and the ranking methods in CA-CondMat.

Based on the above results, we plot the correlation of the influence measures in GDciting, Dolphins and CA-CondMat. The results are shown in Fig 3, Fig 4 and Fig 5 respectively. Due to the large number of nodes in CA-CondMat, we only show the result of the top 500 nodes. We can witness that there is a clear correlation between *F*(*t*) and OC, while the traditional measures, i.e., the BC and the CC, have little relationship with the influence capability of the spreaders in an epidemic process.

**Fig 6:**
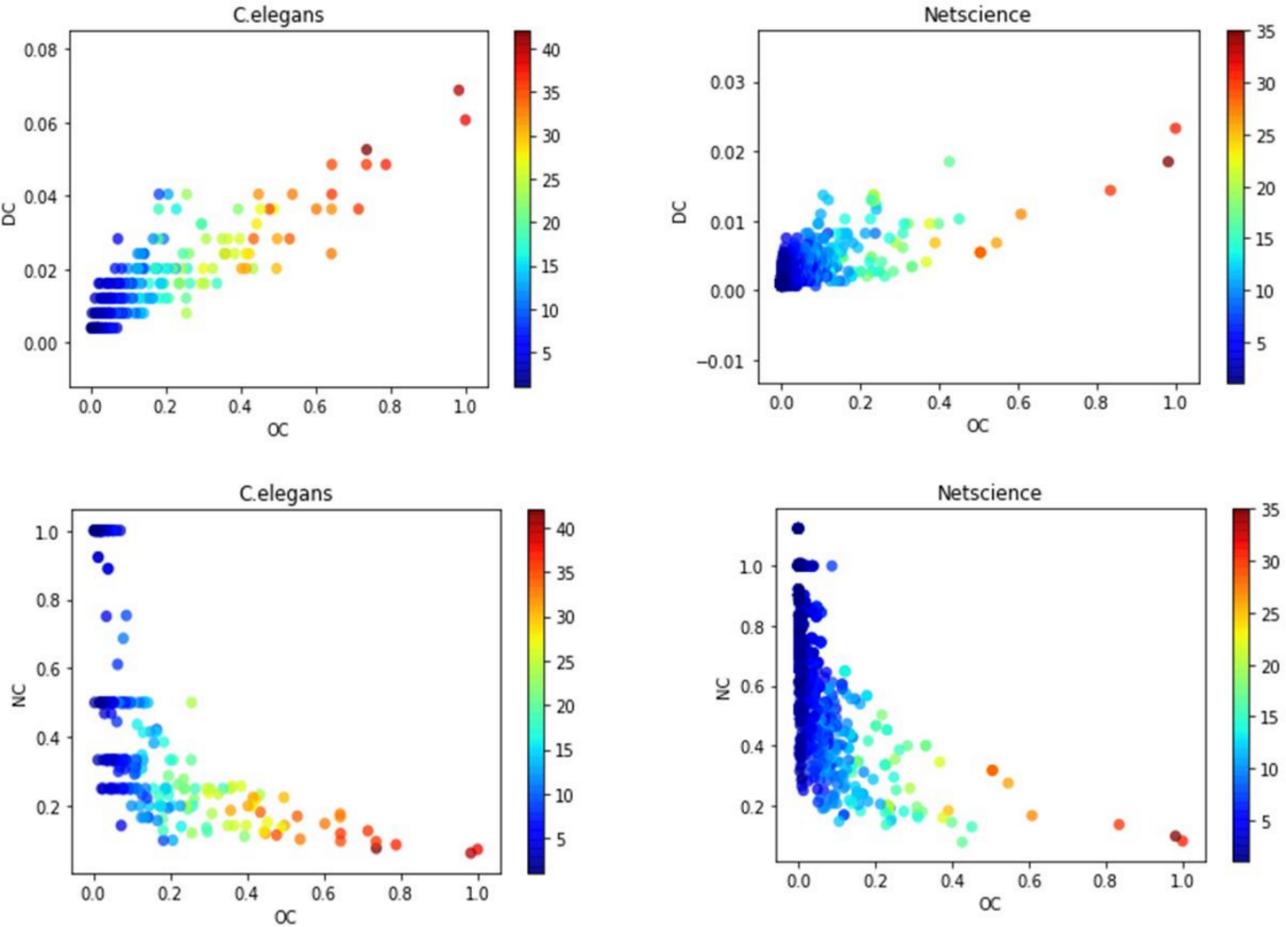
The relations between OC and NC, OC and DC in Netscience and C.elegans.

As the proposed method OC contains the network constraint coefficient and the node degree information, we compare OC with DC and NC. The results are shown in Fig 6. The color of each point represents the influence of the node. We can observe that OC has strong correlation with DC and NC, but there are still many influential nodes with small values of DC and many little influential nodes with small values of NC. It indicates that NC or DC alone is not sufficient to identify influential nodes. The proposed method OC contains both constraint coefficient and node degree information. It can identify influential nodes better.

**Fig 7:**
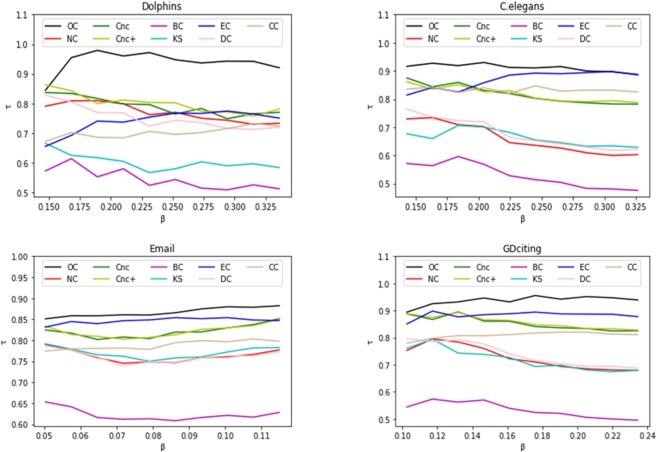
The rank correlation coefficient, Kendall’s tau τ, is plotted by varying the infection probability β in four networks: Dolphins, C.elegans, Email and GDciting.

**Fig 8:**
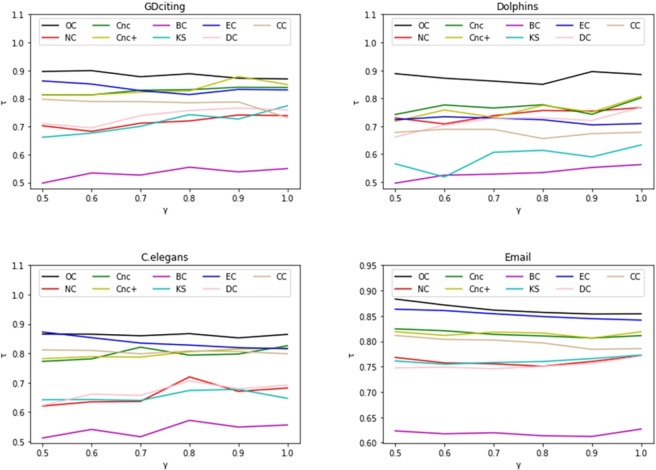
The rank correlation coefficient, Kendall’s tau *τ*, is plotted by varying the recovery probability *γ* in four networks: GDciting, Dolphins, C. elegans, Email.

To further estimate how the infection probability *β* affects the effectiveness of different methods, the Kendall correlation coefficient *τ* as a function of *β* for different methods is shown in Fig 7. The infection probability *β* varies from *β*_*th*_ to 2*β*_*th*_. As described in Fig 7, on a wide range of probabilities *β*, OC is better than other measures in the four networks. In Fig 8, we conduct the same experiments for different values of *γ*, which varies from 0.5 to 1. As shown in Fig 8, the proposed OC presents better results than the other measures in the four networks.

**Fig 9:**
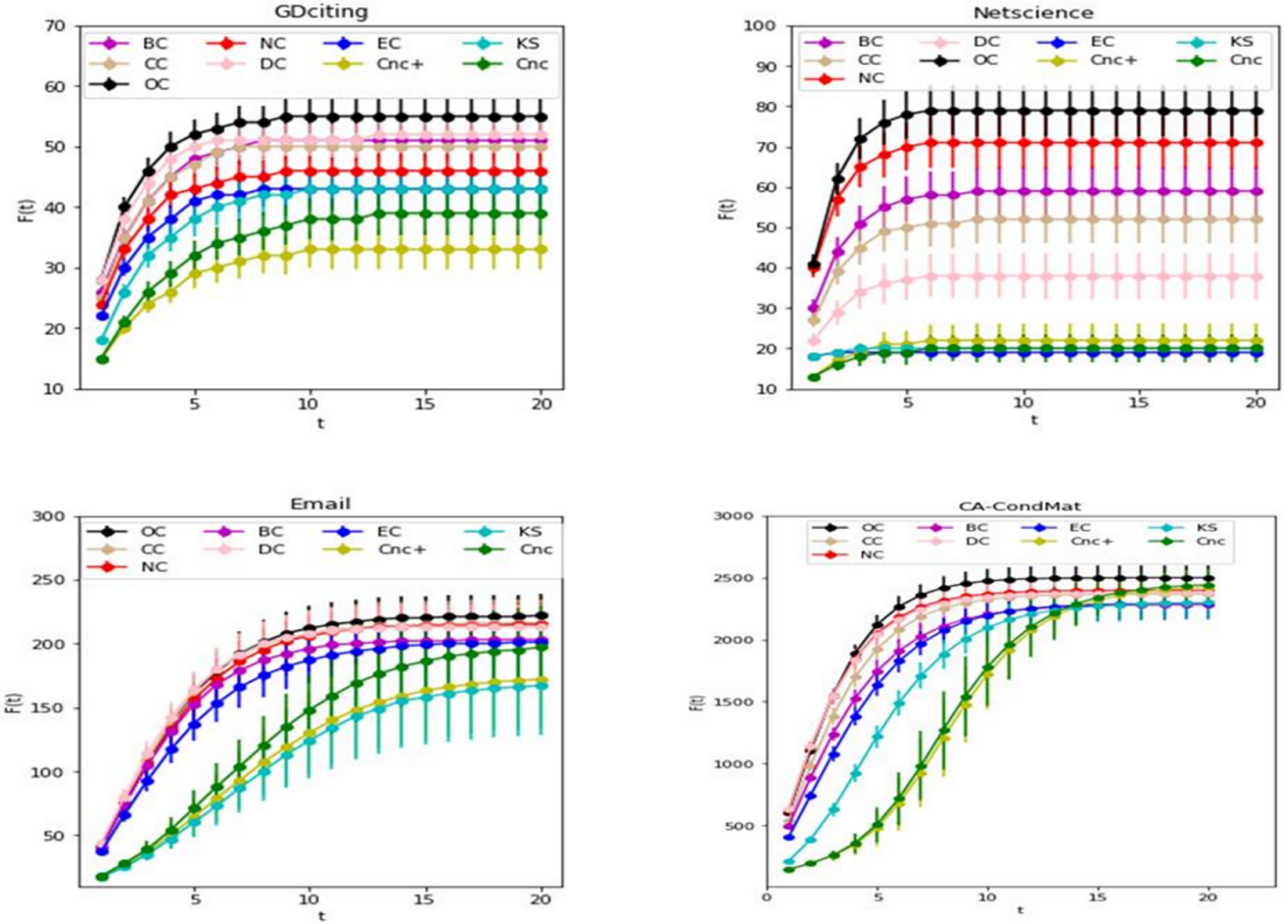
The cumulative number of infected nodes as a function of time *t* in four networks: GDciting, Netscience, Email and CA-CondMat.

Furthermore, we investigate the number of nodes being at the infected and recovered state for various timesteps of the SIR model. We focus on the nodes that appear in the top-*k* lists of each method. We initially set these nodes to be infected. Here we set *k* = 10 for the small networks (GDciting, Netscience and Email) and *k* = 100 for the large network (CA-CondMat). The cumulative number of infected nodes (*F*(*t*)) as a function of time *t* in the four networks are shown in Fig 9. Due to the randomness of transmission, the experimental result of SIR model is different in each experiment. We use the error bar graph to present the results. As shown in Fig 9, the number of cumulative infected nodes increases with time and ultimately reach the steady value. For all these four networks, OC outperforms the other methods for both spreading rate and the number of infected nodes.

**Fig 10:**
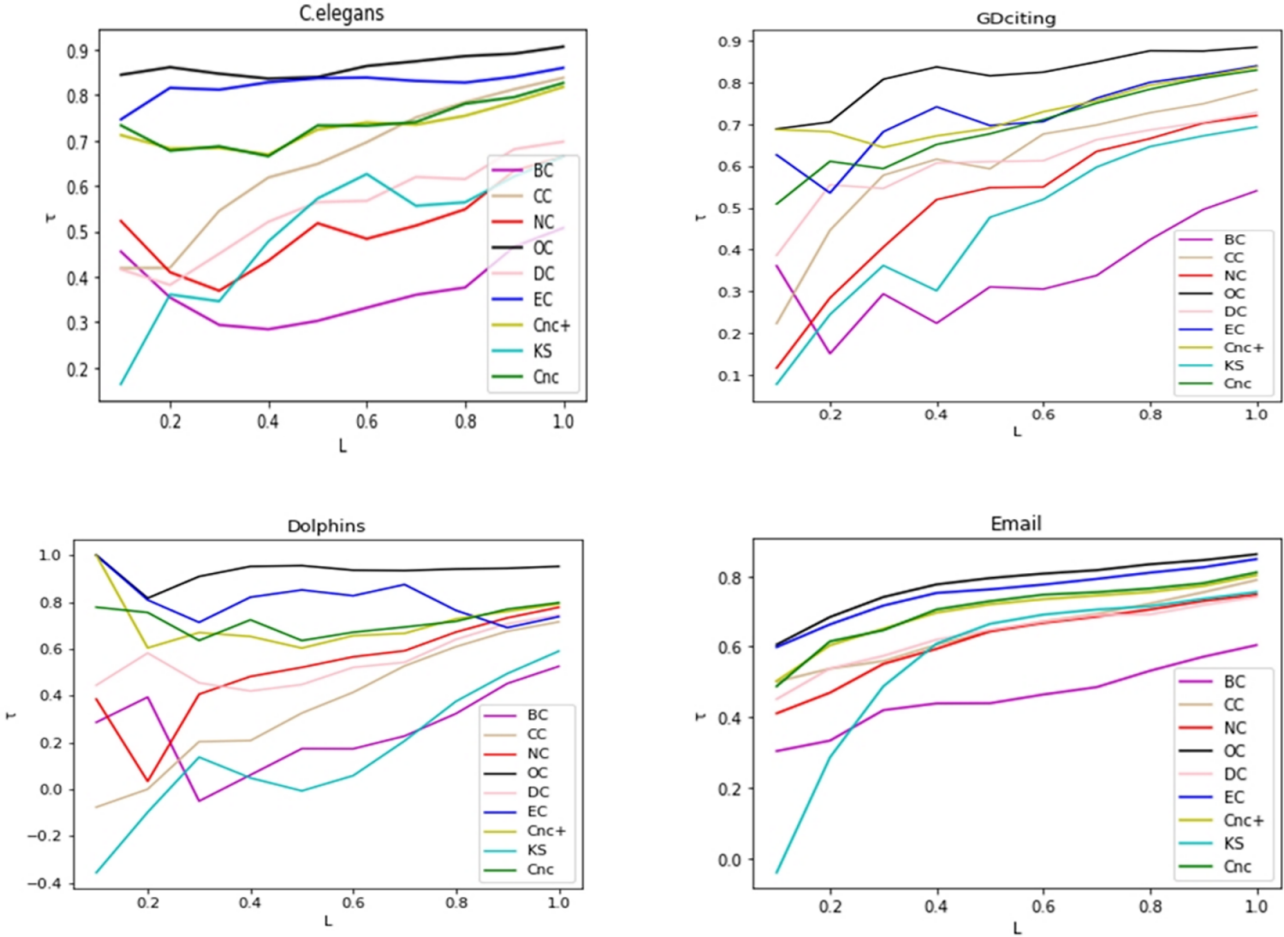
The values of τ for all methods in four networks when *L* varies from 0.1 to 1.0. Here *L* represents the percentage of nodes with the largest spreading ability.

In many cases, people are more interested in a small fraction of the most influential nodes in the network. Here, we use *L* to represent the fraction of the most influential nodes measured by each method. We let *L* vary from 0.1 to 1.0 and do the influence comparison experiment between the top nodes ranked by different methods. As shown in Fig 10, our method outperforms the other methods on almost the entire range of *L* in the four networks.

**Fig 11:**
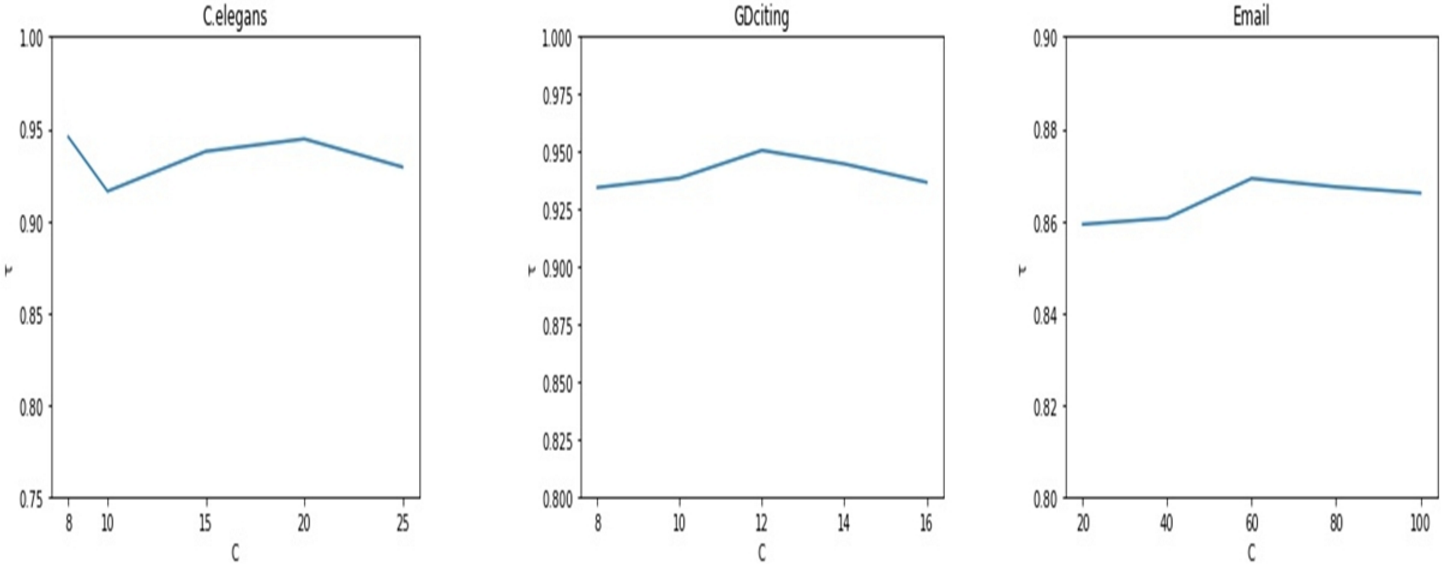
The rank correlation coefficient, Kendall’s tau *τ*, is plotted by varying the number of communities divided in three networks: C.elegans, GDciting and Email.

Determine the number of communities *c* is a challenging task in community detection. As our method need to use the result of community detection, it is necessary to evaluate the impact of the number of communities *c* on the result. We divide the network into different numbers of communities and then identify the influential nodes. Fig 11 shows the results in the three networks. The horizontal axis represents the number of communities divided and the vertical axis is the network correlation coefficient *τ*. As can be seen from Fig 11, the number of communities divided has a limited effect on the results. The fluctuation of *τ* does not exceed 0.1 with *c* varying.

## 4. Conclusion

Identifying influential nodes in complex networks is very important in theoretical and practical applications. In this paper, we proposed an efficient method based on BIGCLAM model. The method suggests that the community overlaps play the "bridging" role between the communities. The number of communities that a node belongs to represents its propagation capacity. In addition, we consider the network constraint coefficient of the node, which represents its propagation speed in community. The comparison results between the proposed method and the benchmark algorithms demonstrated that proposed method can obtain the best results. Our results could shed some light on how to utilize network representation learning and overlapping community detection to identify influential nodes.

## Supporting information

S1 Dataset Nine real-world networks used in this paper. (ZIP)
